# Immunomodulatory Fibrous Scaffold with Dual Enzyme‐Mimic Activities Prevents Postsurgical Tumor Recurrence

**DOI:** 10.1002/advs.202519431

**Published:** 2025-12-27

**Authors:** Xiaoyi Zhao, Zhuolong Jiao, Yue Wang, Huimeng Gu, Longfei Li, Jian Song, Chen Xu, Jiajia Xue, Fu‐Jian Xu, Nana Zhao

**Affiliations:** ^1^ State Key Laboratory of Chemical Resource Engineering Key Laboratory of Biomedical Materials of Natural Macromolecules (Beijing University of Chemical Technology Ministry of Education) Beijing Laboratory of Biomedical Materials College of Materials Sciences and Engineering Beijing University of Chemical Technology Beijing China; ^2^ Beijing Laboratory of Biomedical Materials State Key Laboratory of Organic‐Inorganic Composites College of Materials Sciences and Engineering Beijing University of Chemical Technology Beijing China

**Keywords:** electrospun, immune niche, immunomodulation, implantable scaffold, postsurgical tumor treatment

## Abstract

Surgical resection remains the frontline treatment for solid tumors. However, postsurgical recurrence driven by residual tumor cells and an immunosuppressive microenvironment continues to challenge long‐term survival. Here, we propose an implantable fibrous scaffold functionalized with MnO*_x_* nanozymes to prevent tumor recurrence. The dual enzyme‐mimic activities of the MnO*_x_* nanozymes endow the scaffold with immunomodulatory functions. On one hand, the MnO*_x_* nanozymes with peroxidase‐like activity catalyze the formation of cytotoxic hydroxyl radicals to trigger immunogenic cell death and antigen release. On the other hand, the catalase‐like activity helps alleviate hypoxia by decomposing H_2_O_2_ to O_2_, thereby reprogramming macrophages toward a pro‐inflammatory M1 phenotype. Concurrently, the released Mn^2+^ ions serve as a potent immune adjuvant, promoting immune cell recruitment and activation. The coordinated immunomodulatory cascade enables the scaffold to establish a sustained local immune niche. Both in vitro and in vivo evaluations confirm that this MnO*_x_*‐functionalized scaffold elicits robust antitumor immunity, effectively preventing postsurgical recurrence without exogenous immunostimulants. This work highlights a material‐based immunotherapeutic strategy that leverages the intrinsic catalytic and immunomodulatory properties of nanozymes to engineer bioactive scaffolds for postoperative cancer treatment.

## Graphical abstract

A MnO_x_ nanozyme‐functionalized electrospun fibrous scaffold is developed for postoperative therapy by leveraging its intrinsic physicochemical and immunomodulatory properties. Integrating POD‐ and CAT‐like activities, the scaffold induces immunogenic cell death, alleviates hypoxia, and reverses immunosuppression, while simultaneously recruiting and activating immune cells to stimulate robust antitumor immune responses and prevent postsurgical tumor recurrence.

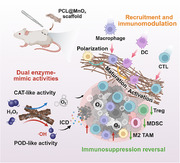

## 1 Introduction

Cancer remains a significant threat to human health worldwide and is still one of the leading causes of mortality [1]. Surgery is still the preferred clinical strategy for most patients with solid tumors [2]. However, a high rate of tumor recurrence occurs after surgery [3]. Cancer immunotherapy has been extensively developed for postsurgical tumor treatment and was considered effective in inhibiting cancer recurrence and metastasis [4]. However, severe local immunosuppression after surgical resection further increases the incidence of relapse and metastases by promoting cancer cell invasion and suppressing the activity of antitumor immune cells, which greatly hampers the therapeutic efficacy [5, 6]. Therefore, the attempts to effectively modulate postsurgical immunosuppression are urgently required. Hydrogels [5, 7, 8], implantable scaffolds [9, 10, 11, 12, 13, 14, 15], and microneedles [16, 17] have been developed for postsurgical tumor treatment and immunomodulation due to their convenient administration and excellent biocompatibility. Among them, implantable scaffolds can be utilized to fill the resected cavity, minimize side effects, and realize localized delivery of the encapsulated cargoes [9, 10, 13, 15]. These scaffolds hold great potential, particularly for breast cancers, which require postsurgical filling [15]. Furthermore, implantable scaffolds with porous structure have been reported to facilitate the immune cell recruitment and infiltration [13, 18, 19, 20, 21]. Typically, these implantable scaffolds are loaded with immune stimulants such as antigens [13, 18], adjuvants [12, 18, 21], and chemokines [18, 22] to enhance antitumor immunity, which is costly and complicated. On the other hand, the intrinsic physicochemical properties, as well as the immunomodulatory functions of implantable scaffolds, remain to be explored. Therefore, it would be desirable to develop implantable scaffolds with inherent properties to eliminate residual tumor and activate antitumor immunity to inhibit postsurgical recurrence.

Electrospun fibrous scaffolds have attracted considerable attention in tumor treatment owing to their high surface area, porosity, and structural similarity to the native extracellular matrix [23, 24]. Due to their nanoscale diameter, electrospun fibers possess a high surface area, which facilitates cell adhesion, proliferation, and migration [25]. Various functional components, such as drugs [26, 27, 28, 29], proteins [30], and nanoparticles [31, 32, 33], have been integrated into the scaffolds. Blend electrospinning and coaxial electrospinning are commonly employed to load drugs and proteins into the scaffolds [23, 34]. However, nanoparticles may exhibit uneven distribution and inevitable agglomeration within the spinning solution. Surface functionalization of electrospun fibrous scaffolds with nanoparticles represents an ideal approach, as it not only increases the nanoparticle bioavailability but also leverages the scaffold architecture to facilitate immune cell recruitment and local immune modulation. Manganese oxide (MnO*_x_*) nanomaterials are attractive candidates in tumor therapy [35, 36]. Due to the mixed‐valence states of Mn, MnO*_x_* nanomaterials could serve as nanozymes with catalase (CAT)‐, peroxidase (POD)‐, and oxidase‐like activities [37]. Moreover, the characteristic tumor microenvironment (TME), featuring mild acidity, elevated hydrogen peroxide (H_2_O_2_) levels, and overexpressed glutathione (GSH), allows MnO*_x_* nanomaterials to decompose H_2_O_2_ to generate O_2_ through CAT‐like activity to alleviate tumor hypoxia, thereby ameliorating immunosuppression [37, 38, 39]. Meanwhile, MnO*_x_* nanomaterials can be gradually degraded into Mn^2+^ in the TME [37, 40], which can further generate toxic hydroxyl radicals (·OH) via POD‐like activity to induce immunogenic cell death (ICD) and provoke antitumor immune responses [37, 41, 42]. Additionally, MnO*_x_* has been shown to promote dendritic cell (DC) maturation and macrophage polarization toward M1 phenotype [37, 38, 39, 42, 43], making it an ideal candidate for endowing scaffolds with intrinsic immunomodulatory properties. Recently, MnO_2_‐functionalized electrospun fibrous scaffolds have been developed to prevent postsurgical adhesion [44]. But the inherent antitumor therapeutic effects and immunomodulatory properties of the scaffold have not been explored. Compared with MnO_2_, MnO*_x_* with mixed valence states creates a more dynamic active center. The unpaired e_g_ electrons in Mn^2+^ and Mn^3+^ ions are crucial for facilitating efficient electron transfer to H_2_O_2_ and subsequent radical generation [45], which may enable enhanced multi‐enzyme mimicry and rapid release of immunostimulatory Mn^2+^ in the TME. Therefore, MnO*_x_*‐functionalized electrospun fibrous scaffolds are ideal to enhance interactions with immune cells, thereby eliciting robust antitumor responses.

**FIGURE 1:**
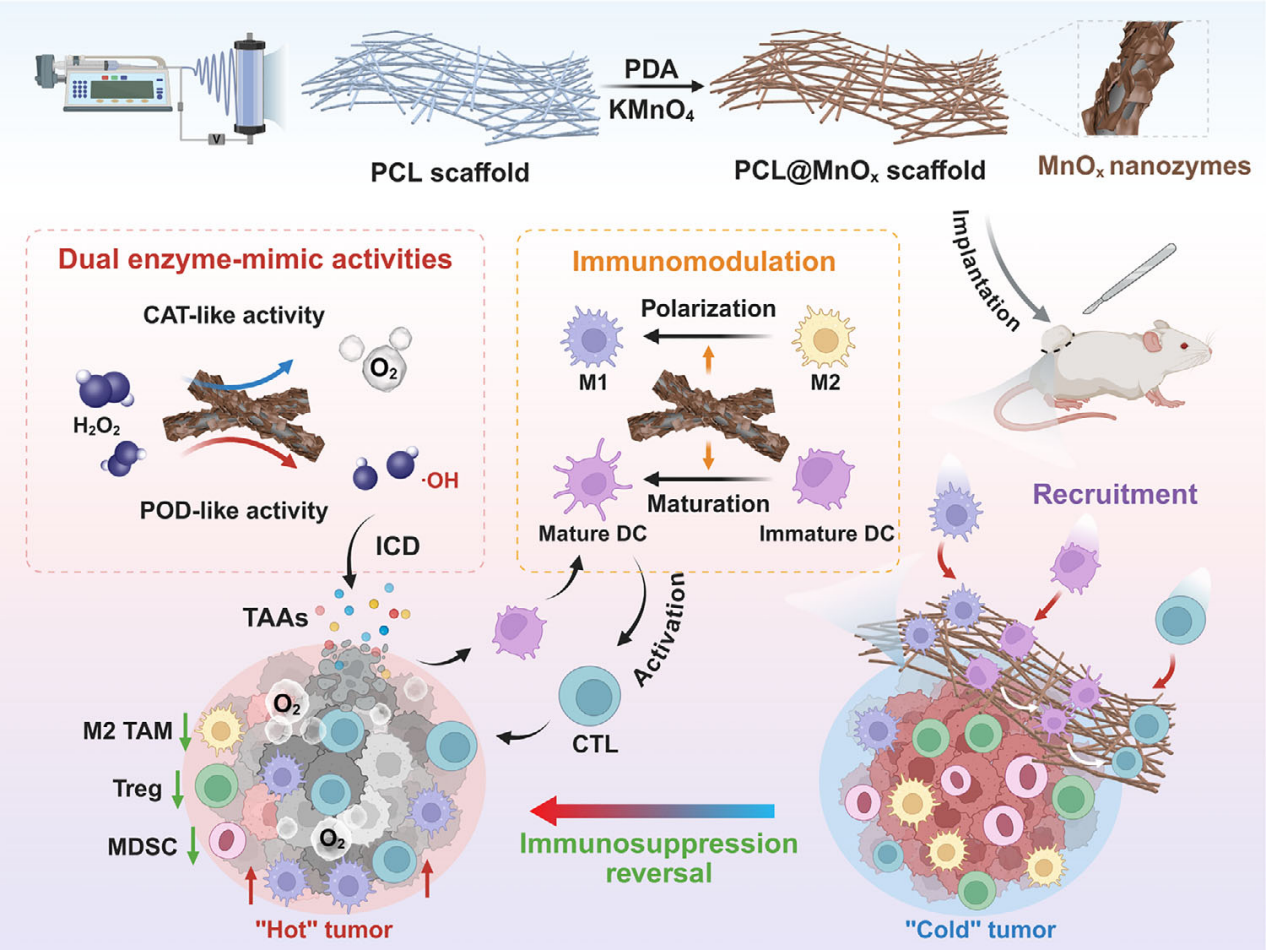
Schematic illustration of the preparation of the PM scaffold for the recruitment and activation of immune cells and inhibition of postsurgical tumor recurrence. (Created with BioRender.com).

Herein, we propose the rational design of MnO*_x_*‐functionalized electrospun fibrous scaffolds (PCL@MnO*_x_*, PM) with dual enzyme‐mimic activities for the suppression of postsurgical tumor recurrence (Figure 1). POD‐like activity of PM scaffolds catalyzes the generation of toxic ·OH, which could induce ICD and promote the release of tumor‐associated antigens (TAAs). The released TAAs, together with the porous structure of the scaffolds, enable the recruitment of immune cells, including macrophages, DCs, T cells, and natural killer (NK) cells. Within the scaffold microenvironment, TAAs and Mn^2+^ adjuvants synergistically activate antigen‐specific immune cells to eradicate tumor cells. Meanwhile, the CAT‐like activity of PM scaffolds decomposes H_2_O_2_ into O_2_, thereby alleviating tumor hypoxia and reprogramming macrophages toward an M1 phenotype to reverse immunosuppression. The feasibility of PM scaffolds to harness their intrinsic physicochemical properties for eliciting potent antitumor immunity and suppressing postsurgical tumor recurrence is comprehensively demonstrated through in vitro and in vivo studies.

## 2 Results and Discussion

### 2.1 Preparation and Characterizations of Fibrous Scaffolds

**FIGURE 2:**
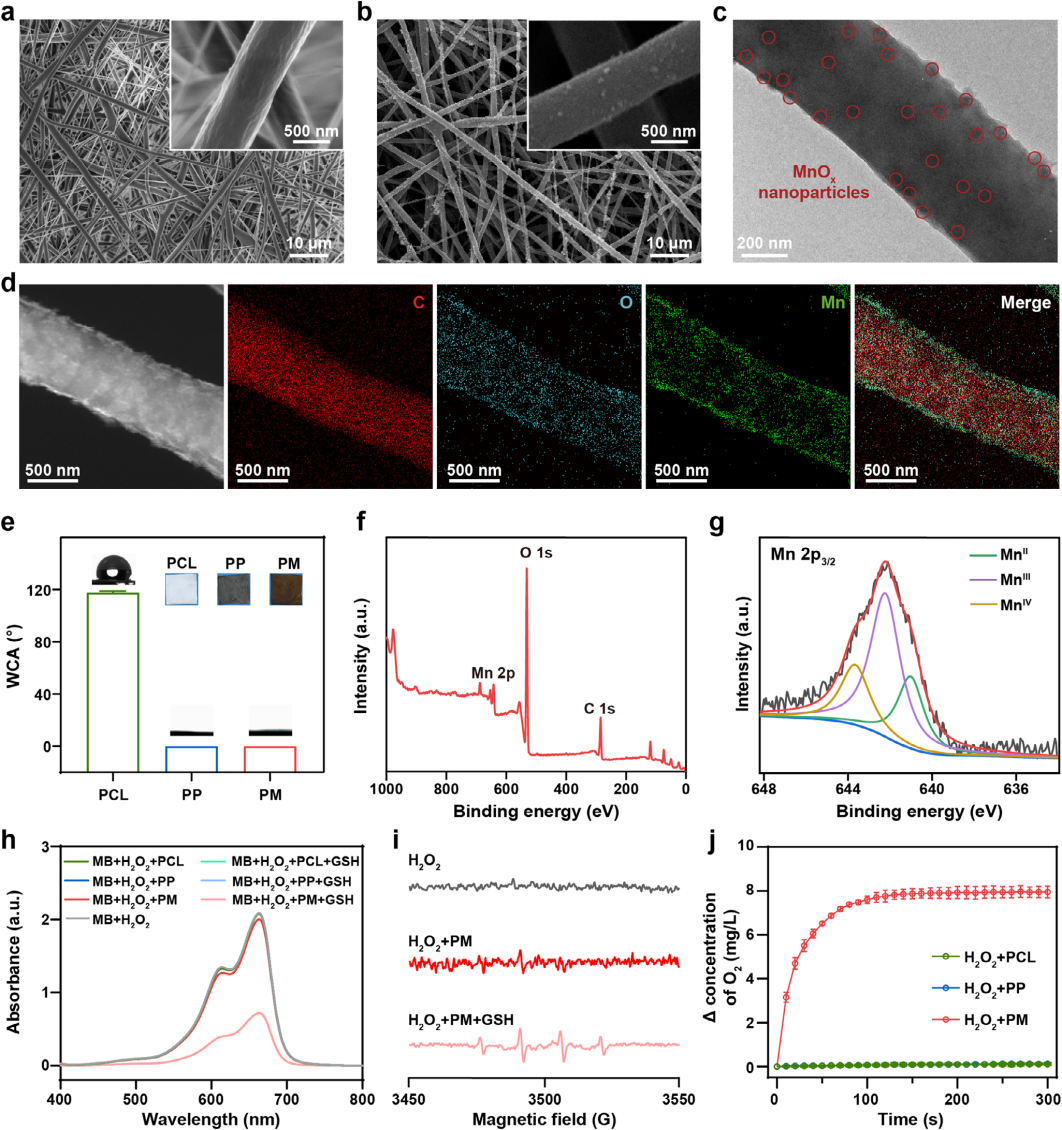
Characterization of PM fibrous scaffolds. SEM images of (a) PCL and (b) PM scaffolds. (c) TEM image of PM fiber. Red circles indicate MnO*x* nanoparticles on the surface of the fiber. (d) Scanning TEM image and the corresponding element mapping images of the PM fiber. (e) The water contact angles of different scaffolds (mean ± SD, *n* = 3). Insets show the photographs of the scaffolds. (f) Full‐range survey XPS spectrum and (g) Mn 2p XPS spectrum for PM scaffold. (h) UV–vis spectra of MB treated by scaffolds under different conditions (MnO*_x_*: 30 µg mL^−1^). (i) ESR spectra of ·OH trapped by DMPO in different samples (MnO*_x_*: 30 µg mL^−1^). (j) Time‐dependent O_2_ concentration changes in H_2_O_2_ solution treated by various scaffolds (MnO*_x_*: 30 µg mL^−1^, mean ± SD, *n* = 3).

Poly(*ε*‐caprolactone) (PCL) fibrous scaffold was first prepared via electrospinning. The MnO*_x_*‐functionalized PCL (PM) fibrous scaffold was subsequently fabricated by modification of the PCL scaffold with polydopamine (PDA) to form the PCL@PDA (PP) fibrous scaffold, which was employed to reduce KMnO_4_ and immobilize MnO*_x_* in situ. It is speculated that low‐valence Mn ions (Mn^2^⁺ and Mn^3^⁺) exhibit POD‐like and oxidase‐like activities. Meanwhile, high‐valence Mn⁴⁺ possesses CAT‐like activity and imparts the ability to deplete GSH [37]. The morphology of the PCL and PM fibers was characterized by scanning electron microscopy (SEM), as displayed in Figure 2a,b. The PCL fibers exhibited a relatively smooth surface, while increased surface roughness could be observed after MnO*_x_* functionalization. PM fiber was further examined by transmission electron microscopy (TEM), demonstrating MnO*_x_* nanoparticles with a diameter of ∼27 nm (Figure 2c). Elemental mapping in Figure 2d demonstrated the uniform distribution of carbon (C) and oxygen (O) elements throughout the fiber, and manganese (Mn) element in the shell, confirming the successful functionalization of MnO*_x_* on the surface of the PM fiber. As displayed in Figure S1, the resultant PM scaffold showed a high Brunauer–Emmett–Teller surface area of ∼39.8 m^2^/g and a total pore volume of ∼0.19 cm^3^/g, verifying the porous structure of the scaffold. The PCL scaffold was found to exhibit a water contact angle (WCA) of ∼117.6°, indicating its hydrophobicity. In contrast, the WCA of PM scaffolds was close to 0°, suggesting a complete transition to hydrophilic surfaces, which was expected to enhance cell attachment and immune cell recruitment.

To investigate the chemical composition of MnO*_x_* on the PM scaffold, the X‐ray photoelectron spectroscopy (XPS) spectrum was conducted. The full‐range survey of the XPS spectrum revealed the presence of Mn and O elements in the PM scaffold (Figure 2f). Notably, the high‐resolution spectrum of Mn 2p demonstrates the presence of Mn in mixed valence states, with characteristic peaks corresponding to Mn^II^, Mn^III^, and Mn^IV^, respectively (Figure 2g). In addition, Mn^III^ is the dominant species with a relative intensity of ∼54%. The content of MnO*_x_* nanozyme in the PM scaffold was determined to be ∼5.6 wt.% by inductively coupled plasma mass spectrometry. It is known that MnO_x_ with mixed valence states displays multienzyme‐like activity and GSH‐depletion capability, which can release Mn^2+^ with POD‐like activity to decompose H_2_O_2_ and generate ·OH [37, 40]. To further investigate the degradation behavior of the MnO*_x_* nanozyme within the scaffold, PM scaffolds were immersed in PBS solution (pH 7.4) in the presence or absence of GSH. As shown in Figure S2, nearly 100% of MnO*_x_* was degraded within 14 d in the presence of GSH, verifying the good degradability of the nanozyme. At the same time, a relatively small weight loss (∼5.8%) was observed for the PM scaffold over 14 d in the presence of GSH, indicating the slow biodegradability kinetics of the scaffold. To validate the POD‐like activity and GSH‐depletion capability of the PM scaffolds, methylene blue (MB) was employed as an indicator. As exhibited in Figure 2h, the PM scaffolds effectively generated reactive oxygen species (ROS) in the presence of both H_2_O_2_ and GSH, verifying the enhanced Mn^2+^ ions release in the TME. In contrast, negligible ROS was detected in the PCL or PP scaffolds control groups, confirming that the POD‐like activity originated from MnO_x_ nanoparticles. The electron spin resonance (ESR) spectra further confirmed the generation of ·OH, displaying a characteristic quartet signal of 1:2:2:1 using DMPO (5,5‐dimethyl‐1‐pyrroline N‐oxide) as the trapping agent (Figure 2i). The CAT‐like activity of the scaffolds was studied employing a dissolved oxygen meter. Compared with the PCL and PP scaffolds, obvious O_2_ release was observed in H_2_O_2_ solution after incubation with PM scaffolds (Figure 2j), demonstrating their potential to alleviate hypoxia in the TME. Therefore, the MnO*_x_* nanozymes provide the PM scaffold with dual enzyme‐mimic activities. Taken together, MnO*_x_* nanozyme‐functionalized fibrous scaffolds hold great potential for ·OH production and O_2_ generation in the TME.

### 2.2 Immunomodulatory Effects of PM Scaffolds In Vitro

**FIGURE 3:**
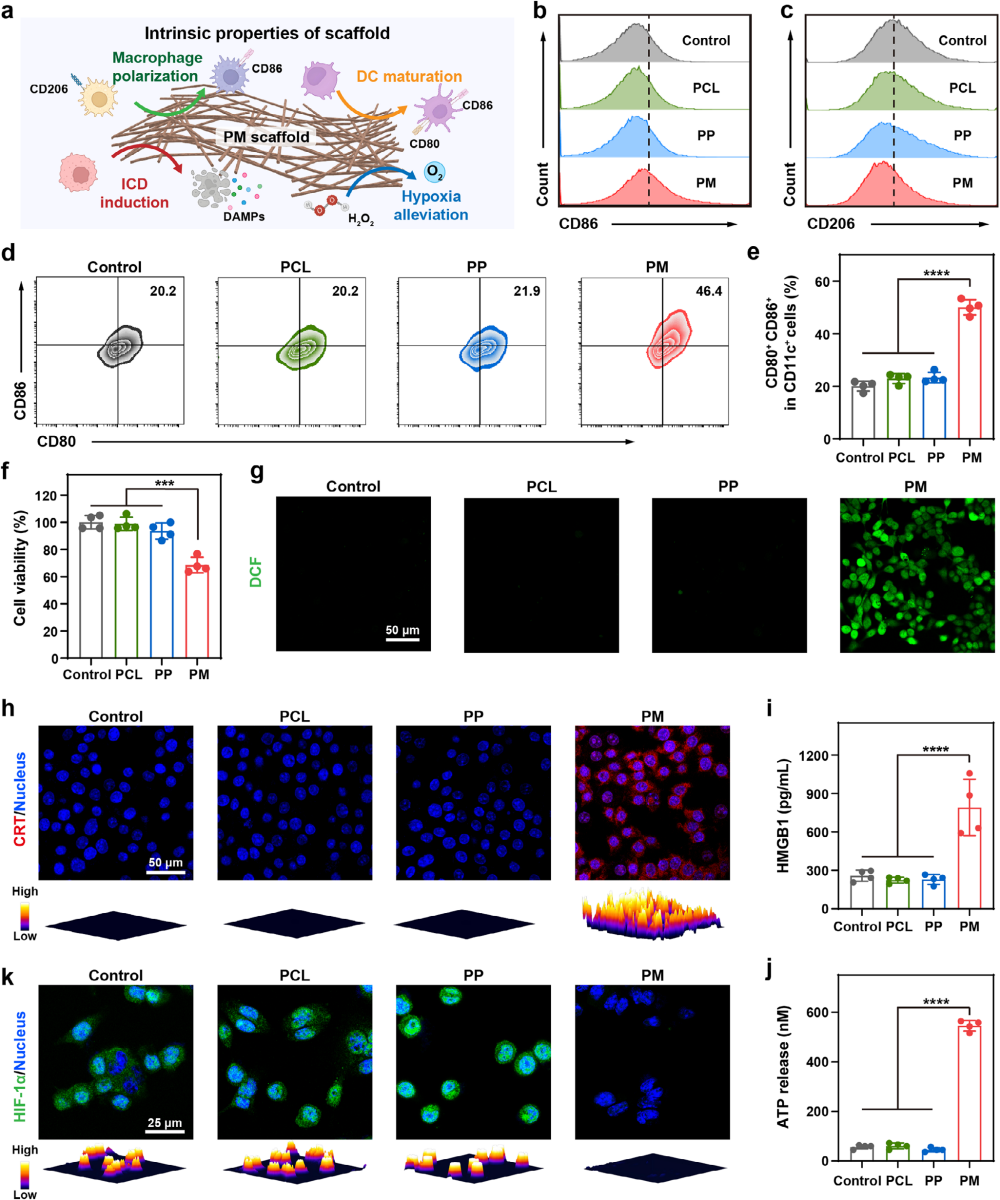
Immunomodulatory effects of PM scaffolds in vitro. (a) Schematic illustration of immunomodulation, ICD induction, and hypoxia alleviation mediated by PM scaffold. (Created with BioRender.com). Flow cytometric analysis of (b) CD86 (M1 surface marker) and (c) CD206 (M2 surface marker) on RAW264.7 cells after incubation with various scaffolds. (d) Representative flow cytometric analysis and (e) quantification of mature DCs (CD80^+^CD86^+^ in CD11c^+^ cells) after incubation with various scaffolds (mean ± SD, *n* = 4). (f) Viability of 4T1 cells after incubation with various scaffolds (mean ± SD, *n* = 4). (g) Representative CLSM images of intracellular ROS production after treatment with various scaffolds. (h) Representative CLSM images with quantified 3D surface plots for visualizing CRT exposure after different treatments. Extracellular release of (i) HMGB1 and (j) ATP from 4T1 cells after different treatments (mean ± SD, *n* = 4). (k) Representative CLSM images with quantified 3D surface plots of HIF‐1*α* expression of 4T1 cells after different treatments. MnO*_x_*: 150 µg mL^−1^. One‐way ANOVA followed by Tukey's test. ^***^ *p*< 0.001, and ^****^ *p*< 0.0001.

Macrophages and DCs play critical roles in immunomodulation and initiation of antitumor immune responses. Considering the ability of MnO_x_ nanomaterials to alleviate hypoxia, deplete GSH, and release Mn^2+^ in the TME [38], which have been reported to elicit antitumor immunity by stimulating macrophage M1 polarization and DC maturation [37, 39, 40, 41, 42], we further evaluated the immunoregulatory functions of PM scaffolds (Figure 3a). Biocompatibility represents a critical prerequisite for further biomedical applications. Therefore, the cytotoxicity of the scaffolds was first evaluated. As displayed in Figure S3, the viability of L929, RAW264.7, and DC2.4 cells treated with PCL, PP, and PM scaffolds remained above 80%. Subsequently, the ability of the scaffolds to promote macrophage M1 polarization was evaluated by flow cytometry. Interleukin 4 (IL‐4)‐pretreated RAW264.7 macrophages (M2 phenotype) were incubated with PCL, PP, and PM scaffolds, respectively. As shown in Figure 3b and Figure S4a, PCL and PP scaffolds exhibited negligible influence on the polarization of M2 macrophages to the M1 phenotype. In contrast, the proportion of M1 macrophages was upregulated to ∼35.0% after the PM scaffold treatment. Furthermore, PM scaffolds effectively reduced the population of M2 macrophages to ∼34.2%, compared with the PCL (∼49.6%) and PP (∼48.1%) counterparts (Figure 3c; Figure S4b). These results indicate that PM scaffolds exhibited superior abilities in polarizing macrophages. The ability of scaffolds to stimulate DC maturation was further evaluated by analyzing the proportion of mature DCs after incubation with different scaffolds (Figure 3d,e). An obvious increase in mature DCs was observed after treatment with PM scaffolds, confirming the immunoadjuvant effect of the MnO*_x_*‐functionalized scaffolds. Collectively, these results suggest that PM scaffolds possess superior immunoregulatory properties to induce macrophage M1 polarization and DC maturation.

### 2.3 ICD Elicited by PM Scaffolds In Vitro

Inspired by the excellent GSH depletion and ·OH generation performance of MnO*_x_* nanozyme‐functionalized fibrous scaffolds, the antitumor efficacy of PM scaffolds was further investigated. Considering the elevated levels of GSH and H_2_O_2_ in tumor cells compared with normal cells [46, 47], the killing effect of the scaffolds on 4T1 tumor cells was further assessed. As displayed in Figure 3f, PCL and PP scaffolds exhibited minimal killing effect on tumor cells. In contrast, the viability of 4T1 cells treated with PM scaffolds significantly decreased to ∼68.5%. Furthermore, intracellular ROS were detected using confocal laser scanning microscopy (CLSM) with 2,7‐dichlorofluorescein diacetate as a probe. As anticipated, 4T1 cells treated with PM scaffolds displayed obvious green fluorescence signals (Figure 3g), suggesting a significant increase in intracellular ROS levels. These results suggest that PM scaffolds could effectively induce tumor cell death through ROS generation.

The generated ROS has been demonstrated to trigger ICD, leading to the release of TAAs and damage‐associated molecular patterns (DAMPs) to boost antitumor immunity. To evaluate the ability of the scaffolds to induce ICD, surface‐exposed calreticulin (CRT), passively released high mobility group box 1 (HMGB1), and secreted adenosine triphosphate (ATP) were examined. CLSM imaging revealed pronounced CRT exposure on the cell membrane in the PM group compared with the PP and PCL groups (Figure 3h). Meanwhile, PM scaffolds significantly promoted HMGB1 release and ATP secretion (Figure 3i,j). Collectively, these results indicate that PM scaffold treatment can effectively induce ICD. In addition, inspired by the ability of MnO*_x_* nanomaterials to decompose H_2_O_2_ to produce O_2_ [37, 38, 41, 42], the intracellular hypoxia was visualized by the expression of hypoxia‐inducible factor‐1*α* (HIF‐1*α*). As expected, the incubation with PM scaffolds led to a significant downregulation of HIF‐1*α* expression (Figure 3k). Taken together, these results suggest the potential of PM scaffolds to alleviate tumor hypoxia and polarize macrophages from protumorigenic M2 to the antitumor M1 phenotype, which might facilitate the tumor‐killing activity of T cells [48].

### 2.4 PM Scaffolds for the Suppression of Postsurgical Tumor Recurrence

**FIGURE 4:**
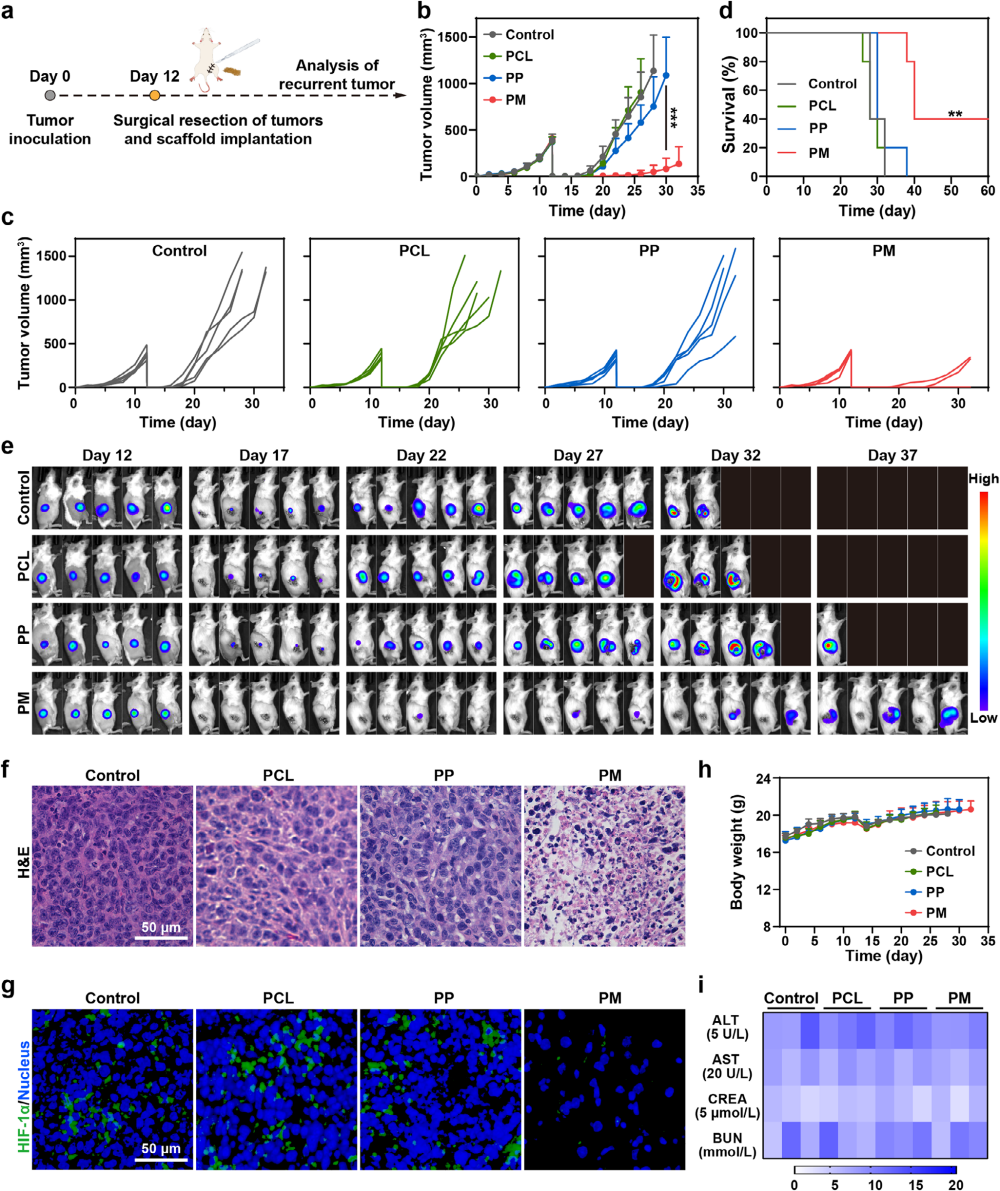
PM scaffolds for suppression of postsurgical tumor recurrence. (a) Schematic illustration of the scaffold‐mediated therapy to inhibit tumor recurrence in the 4T1‐Luc postsurgical tumor model. (b) Average and (c) individual tumor growth curves in different groups (mean ± SD, *n* = 5). (d) Survival curves of the mice treated with different treatment groups (*n* = 5). (e) Bioluminescence images of mice after varied treatments as indicated (*n* = 5). (f) H&E staining images of tumors after different treatments. (g) Immunofluorescence staining of tumor tissue sections with HIF‐1*α* staining following different treatments. (h) Weight changes of mice in different groups (mean ± SD, *n* = 5). (i) Blood biochemical parameters of mice with different treatments (*n* = 3). MnO*_x_*: 675 µg per mouse. One‐way ANOVA followed by Tukey's test. ^**^ *p*< 0.01, and ^***^ *p*< 0.001.

Motivated by the superior immunomodulation and antitumor performance of PM scaffolds in vitro, we further investigated their therapeutic efficacy in a mouse tumor resection model (Figure 4a). Female Balb/c mice were subcutaneously inoculated with 4T1‐Luc cells. On day 12 after inoculation, visible tumors reached a volume of approximately 300 mm^3^. Subsequently, the tumors were resected, and scaffolds were implanted into the resection site. The tumor volume and body weight were measured and recorded every other day. Tumor growth curves in Figure 4b,c demonstrate a significant suppression of tumor recurrence following PM scaffold implantation, whereas PCL and PP scaffolds exhibited minimal antitumor efficacy, consistent with the in vitro findings. Survival curves (Figure 4d) show that mice implanted with PM scaffolds maintained an evidently high survival rate. 40% of the mice after PM treatment survived up to 60 days, indicating that PM scaffolds could effectively inhibit postsurgical tumor recurrence. Tumor growth was also monitored by bioluminescence signals from 4T1‐Luc cancer cells (Figure 4e; Figure S5). Bioluminescence images reveal that PCL and PP scaffolds had negligible effects on postsurgical tumor suppression, in agreement with the tumor growth curves (Figure 4b,c). Encouragingly, PM scaffolds exhibited potent inhibition against tumor recurrence. In addition, histological analysis of tumors further confirmed the therapeutic efficacy, with the PM group exhibiting the most severe cell necrosis and apoptosis (Figure 4f). Considering the satisfactory ability of PM scaffold to generate O_2_ and alleviate tumor hypoxia in vitro (Figures 2j and 3k), we further evaluated the hypoxia level of the tumor. As exhibited in Figure 4g, an obvious downregulation of HIF‐1*α* was observed in tumors treated with the PM scaffold, which could be beneficial to the modulation of the immunosuppressive TME.

In addition, no significant differences in body weight were observed among different treatments, and no apparent weight loss was observed (Figure 4h). Meanwhile, the implantable scaffolds did not cause any noticeable histopathological abnormalities in major organs (Figure S6). Furthermore, important blood biochemical indicators of liver and kidney function, including alanine aminotransferase (ALT), aspartate aminotransferase (AST), creatinine (CREA), and blood urea nitrogen (BUN), remained within normal levels across all groups (Figure 4i). Taken together, these results demonstrate that PM scaffolds can effectively suppress postsurgical tumor recurrence with excellent biosafety in vivo.

### 2.5 Recruitment and Stimulation of Immune Cells by PM Scaffolds In Vivo

**FIGURE 5:**
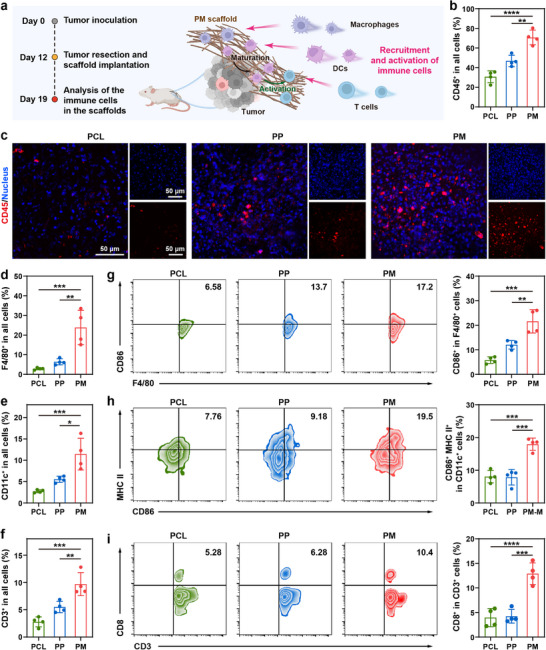
In vivo recruitment and activation of immune cells. (a) Schematic illustration of recruitment and activation of immune cells in the PM scaffold 7 days after implantation. (Created with BioRender.com). (b) Flow cytometric analysis and (c) representative immunohistochemical images of CD45^+^ cells in different scaffolds. Proportion of (d) macrophages (F4/80^+^ cells), (e) DCs (CD11c^+^ cells), and (f) T cells (CD3^+^ cells) in infiltrated cells in different scaffolds 7 days after implantation, analyzed by flow cytometry (mean ± SD, *n* = 4). (g) Flow cytometry analysis of CD86^+^ cells in macrophages in different scaffolds (mean ± SD, *n* = 4). (h) Flow cytometry analysis of mature DCs (CD80^+^CD86^+^ cells in CD11c^+^ cells) in different scaffolds (mean ± SD, *n* = 4). (i) Flow cytometry analysis of CTLs (CD8^+^ cells in T cells) in different scaffolds (mean ± SD, *n* = 4). One‐way ANOVA followed by Tukey's test. ^*^ *p*< 0.05, ^**^ *p*< 0.01, ^***^ *p*< 0.001, and ^****^ *p*< 0.0001.

Given the efficacy of PM scaffolds in inhibiting postsurgical tumor recurrence in vivo, we aimed to elucidate the underlying mechanisms. The porous structure of the PM scaffold and the TAAs released are supposed to favor immune cell infiltration [13, 18]. To explore the immune cell activation ability of the PM scaffold, immune cell infiltration within the scaffolds was first investigated by flow cytometry and immunofluorescence staining (Figure 5a). A substantially elevated infiltration of immune cells was observed in the PM scaffold compared with PCL and PP scaffolds (Figure 5b,c; Figure S7). On day 7 post‐implantation, approximately 70% of the infiltrating cells in the PM scaffold were CD45^+^ leukocytes (Figure 5b), the majority of which were antigen‐presenting cells (APCs), including macrophages (F4/80^+^, Figure 5d; Figure S8) and DCs (CD11c^+^, Figure 5e; Figure S9). Moreover, a pronounced increase in T cells (CD3^+^, Figure 5f; Figure S10) and NK cells (CD49b^+^, Figure S11) was also observed within the PM scaffolds. The efficient recruitment of immune cells can be attributed to the capacity of the PM scaffolds to kill tumor cells and release TAAs, combined with their porous architecture.

Subsequently, the immunostimulatory effects of the scaffolds in vivo were assessed. As displayed in Figure 5g, the upregulation of CD86 in the PM group indicates effective activation of M1 macrophages. Meanwhile, compared with the PCL and PP counterparts, the PM scaffolds could significantly activate DCs, as evidenced by the upregulated expression of CD86 and MHC II (Figure 5h), confirming their role in promoting DC maturation and enhancing antigen presentation to initiate tumor‐specific immune responses. Moreover, the proportion of CD8^+^ T cells was significantly increased in the PM group (Figure 5i), with ∼12.9% CD8⁺ T cells detected, in contrast to ∼4.0% and ∼4.2% in the PCL and PP groups, respectively, suggesting the amplified antitumor immune response to promote cytotoxic T lymphocyte (CTL) infiltration. This can be attributed to the more mature APCs in the PM scaffolds, which can effectively present antigens to T cells, thereby initiating the CTL proliferation. These results indicate that naïve immune cells recruited by PM scaffolds could be activated by TAAs, with the functionalized MnO*_x_* nanoparticles acting as adjuvants, ultimately fostering antigen‐specific immune responses against tumor cells. Taken together, the implantable PM scaffolds could serve as a potent immunomodulatory platform that effectively recruits and activates APCs and T cells to establish an “artificial tertiary lymphoid structure” microenvironment, thereby amplifying antitumor immunity.

### 2.6 Antitumor Immune Responses In Vivo

**FIGURE 6:**
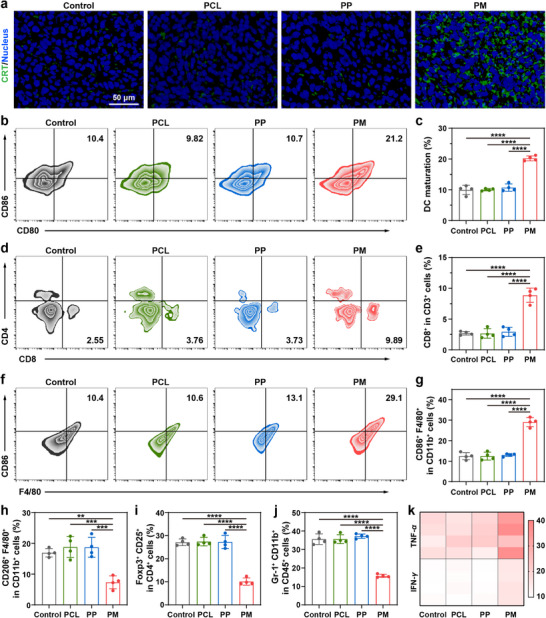
Antitumor immune responses boosted by PM in vivo. (a) Immunofluorescence staining of tumor tissue sections with CRT following different treatments. (b) Representative flow cytometric plots and (c) quantification of mature DCs (CD11c^+^CD80^+^CD86^+^ cells) in lymph nodes (mean ± SD, *n* = 4). (d) Representative flow cytometric plots and (e) quantification of CTLs (CD3^+^CD8^+^ cells) in tumors (mean ± SD, *n* = 4). (f) Representative flow cytometric plots and (g) quantification of M1‐like TAMs (CD11b^+^F4/80^+^CD86^+^ cells) in tumors (mean ± SD, *n* = 4). Flow cytometric analysis of (h) M2‐like TAMs (CD11b^+^F4/80^+^CD206^+^ cells), (i) Tregs (CD3^+^CD4^+^CD25^+^Foxp3^+^ cells), and (j) MDSCs (CD45^+^CD11b^+^Gr‐1^+^ cells) in tumors (mean ± SD, *n* = 4). (k) Expression levels of TNF‐*α* and IFN‐*γ* in the serum measured by ELISA 14 days after implantation (*n* = 4). One‐way ANOVA followed by Tukey's test. ^**^ *p*< 0.01, ^***^ *p*< 0.001, and ^****^ *p*< 0.0001.

Encouraged by the superior capability of PM scaffolds to kill tumor cells and modulate immune cells through recruitment and activation, we then investigated their potential to provoke antitumor immune responses in tumors. Tumors were harvested for immunofluorescence and flow cytometry analyses 14 days after implantation. Since ICD and the release of TAAs promote APC maturation and T cell activation [49], we first assessed the ICD induction in vivo by immunofluorescence staining of CRT. Tumors treated with the PM scaffold exhibited obvious CRT exposure, whereas much weaker fluorescence was observed in other groups (Figure 6a), consistent with the in vitro results (Figure 3h). Moreover, to further elucidate the mechanisms underlying the therapeutic effects, the maturation of DCs in lymph nodes was evaluated (Figure 6b,c). A significantly higher percentage of CD80⁺CD86⁺ DCs (∼20.2%) was detected in the PM group compared to other groups (∼10.0% and 10.7% for PCL and PP groups, respectively), implying that PM scaffolds could effectively promote DC maturation in vivo through immunoadjuvant and ICD induction properties. Furthermore, flow cytometric analysis of tumor tissues revealed a marked increase in the population of intratumoral CD8⁺ CTLs in the PM group (Figure 6d,e). Collectively, the intrinsic properties of PM scaffolds could effectively enhance T cell infiltration and elicitation in the tumor.

The proportion of immunosuppressive cells within the tumor was further analyzed to investigate the regulation of the PM scaffold on the postsurgical immunosuppressive TME. Flow cytometric analysis revealed a considerably increased percentage of M1‐like tumor‐associated macrophages (M1‐like TAMs, CD11b^+^F4/80^+^CD86^+^), accompanied by an evident reduction in protumorigenic M2‐like TAMs (CD11b^+^F4/80^+^CD206^+^) in the PM group (Figure 6f–h). This might be attributed to the effective hypoxia‐alleviating capability of the PM scaffolds through the CAT‐like activity, as tumor hypoxia is supposed to facilitate M2 macrophage polarization [50]. The alleviation of hypoxia is also known to downregulate PD‐L1 expression, thereby ameliorating immunosuppression [51]. Notably, the polarization of macrophages from the M2 to the M1 phenotype was also found to increase the intratumoral H_2_O_2_ concentration [52], which could synergistically enhance the catalytic activity of the PM scaffolds. In addition to TAM reprogramming, PM scaffold implantation led to a significant reduction in the infiltration of immunosuppressive cells, including regulatory T cells (Tregs, CD4⁺CD25⁺Foxp3⁺ cells) and myeloid‐derived suppressor cells (MDSCs, CD45⁺CD11b⁺Gr‐1⁺ cells), compared with the control, PCL, and PP groups (Figure 6i,j). These cells are widely recognized for their roles in inhibiting CTL activity and promoting tumor immune evasion. Taken together, the mechanism and therapeutic effect of PM scaffolds are associated with their dual enzyme‐mimic activities. The POD‐like activity of PM scaffolds facilitates the generation of abundant ·OH to directly kill tumor cells and induce ICD (Figure 6a). Meanwhile, the CAT‐like activity of the PM scaffolds helps relieve tumor hypoxia (Figure 4g), thereby reversing the immunosuppressive TME. At the same time, PM scaffolds effectively recruited APCs and T cells (Figure 5e–i), where the TME‐boosted Mn^2+^ served as a potent adjuvant to promote APC activation (Figure 5f). These activated APCs efficiently presented tumor antigens to T cells, ultimately triggering a robust CTL‐mediated antitumor immune response to prevent postoperative tumor recurrence (Figure 6e). Moreover, the robust antitumor immune responses of PM scaffolds were further validated by elevated serum levels of proinflammatory cytokines tumor necrosis factor‐alpha (TNF‐*α*) and interferon‐gamma (IFN‐*γ*) (Figure 6k), highlighting their immunomodulatory effect. Collectively, these results indicate that PM scaffolds can effectively reshape the immunosuppressive TME by reducing immunosuppressive cell populations, thereby facilitating T cell recruitment, activation, and sustained antitumor immunity to suppress postoperative tumor recurrence.

## 3 Conclusion

In summary, we present a MnO_*x*_ nanozyme‐functionalized electrospun fibrous scaffold as a promising strategy to inhibit postsurgical tumor relapse by harnessing its inherent physicochemical and immunomodulatory properties. The resultant PM scaffold effectively promoted macrophage M1 polarization and DC maturation, while its dual enzyme‐mimic activities enabled the generation of cytotoxic ·OH via POD‐like catalysis to directly eliminate tumor cells and the decomposition of H_2_O_2_ via CAT‐like activity to alleviate hypoxia and reprogram the immunosuppressive TME. Furthermore, the released TAAs and the Mn^2+^ in the TME, together with the porous structure of the scaffolds, facilitate efficient recruitment and activation of immune cells, which collectively contribute to the potent antitumor immunity. Systematic in vitro and in vivo investigations further validated that the MnO_x_‐functionalized scaffolds with dual enzyme‐mimic activities could effectively suppress postsurgical tumor recurrence. This work establishes a new paradigm for implantable biomaterials by demonstrating that scaffolds can be intrinsically engineered to direct multifunctional immune responses, thereby providing a translatable strategy for effective postoperative cancer therapy.

## 4 Experimental Section

### 4.1 Preparation of PCL, PCL@PDA (PP), and PCL@MnO_*x*_ (PM) Scaffolds

The PCL fibrous scaffold was fabricated by electrospinning [53]. The PP scaffold was prepared by decorating the PCL scaffold with a polydopamine layer. The PM scaffold was prepared by the reaction of the PP scaffold with the KMnO_4_ solution. The detailed procedures are described in the Supporting Information.

### 4.2 Cytotoxicity Assay

To evaluate the cytotoxicity of various scaffolds in L929, RAW264.7, DC2.4, and 4T1 cells, the cells were seeded in 24‐well plates followed by incubation with different scaffolds for 24 h. The detailed procedures are described in the Supporting Information.

### 4.3 In Vitro Assessments of Macrophage Polarization and DC Maturation

RAW264.7 cells and BMDCs were seeded in 6‐well plates, respectively. RAW264.7 cells and BMDCs were incubated with different scaffolds. Afterward, the cells were harvested and stained with corresponding antibodies, followed by flow cytometry analysis. The detailed procedures are described in the Supporting Information.

### 4.4 Postsurgical Tumor Models and Anti‐Recurrence Efficacy

Animal experiments were approved by the Animal Ethics Committee of China‐Japan Friendship Hospital (Beijing, China) and performed in accordance with legal protocols. To evaluate the anti‐recurrence efficacy of scaffolds in vivo, 4T1‐Luc cells were subcutaneously inoculated in the right flank of female Balb/c mice on day 0. When the tumor volume reached around 300 mm^3^, mice were randomly divided into four groups: Control, PCL, PP, and PM. Approximately 90% of each tumor was surgically resected, followed by implantation of various scaffolds into the resection cavity. The detailed procedures are described in the Supporting Information.

To study the immune recruitment and stimulation of scaffolds and the immune responses in vivo, scaffolds, tumors, and inguinal lymph nodes were collected for the analysis of immune cells by flow cytometry. The detailed procedures are described in the Supporting Information.

### 4.5 Statistical Analysis

Data are shown as the mean ± SD and are from at least three independent experiments. Statistical significance was calculated by one‐way ANOVA with the Tukey post hoc test. Comparisons of survival rates were calculated by the log‐rank (Mantel‐Cox) test. ^*^ *p*< 0.05, ^**^ *p*< 0.01, ^***^ *p*< 0.001, and ^****^ *p*< 0.0001 were considered as the different statistical significances. All statistical analyses were performed using GraphPad Prism 8 software.

## Conflicts of Interest

The authors declare no conflict of interest.

## Supporting information




**Supporting File**: advs73527‐sup‐0001‐SuppMat.docx.

## Data Availability

The data that support the findings of this study are available from the corresponding author upon reasonable request.
