# Supplementary material for: Immunomodulatory Fibrous Scaffold with Dual Enzyme‐Mimic Activities Prevents Postsurgical Tumor Recurrence
Source: Adv Sci (Weinh). 2025 Dec 27;13(18):e19431. doi: 10.1002/advs.202519431 (PMC13042912; doi:10.1002/advs.202519431)
Supplement: Supplementary file 1 — Supporting File: advs73527‐sup‐0001‐SuppMat.docx. [file ADVS-13-e19431-s001.docx]

Supporting Information

Immunomodulatory Fibrous Scaffold with Dual Enzyme-Mimic Activities Prevents Postsurgical Tumor Recurrence

Xiaoyi Zhao, Zhuolong Jiao, Yue Wang, Huimeng Gu, Longfei Li, Jian Song, Chen Xu, Jiajia Xue,* Fu-Jian Xu,* and Nana Zhao*

X. Zhao, Z. Jiao, Y. Wang, H. Gu, C. Xu, F. J. Xu, N. Zhao

State Key Laboratory of Chemical Resource Engineering

Key Laboratory of Biomedical Materials of Natural Macromolecules (Beijing University of Chemical Technology, Ministry of Education)

Beijing Laboratory of Biomedical Materials

College of Materials Sciences and Engineering

Beijing University of Chemical Technology

Beijing 100029, China

E-mail: xufj@mail.buct.edu.cn; zhaonn@mail.buct.edu.cn

L. Li, J. Song, J. Xue

Beijing Laboratory of Biomedical Materials

State Key Laboratory of Organic-Inorganic Composites

College of Materials Sciences and Engineering

Beijing University of Chemical Technology

Beijing 100029, China

E-mail: jiajiaxue@mail.buct.edu.cn

Experimental Section

*Materials*: Poly(*ε*-caprolactone) (PCL, Mn = 80 kDa) and lipopolysaccharide (LPS) were obtained from Sigma-Aldrich (USA). Dopamine hydrochloride (DA), 3,3',5,5'-tetramethylbenzidine (TMB), tris(hydroxymethyl)aminomethane and 2,2,2-trifluoroethanol (TFE) were obtained from Energy Chemical (China). Potassium permanganate (KMnO_4_), methanol, ethanol, and dichloromethane were purchased from Beijing Chemical Co. (China). (4,5-Dimethylthiazol-2yl)-2,5-diphenyl tetrazolium bromide (MTT) and 4’,6-diamidino-2-phenylindole (DAPI) were purchased from Beijing Solarbio Science & Technology Co., Ltd (China). Murine granulocyte-macrophage colony-stimulating factor (GM-CSF) and murine interleukin 4 (IL-4) were purchased from PeproTech (USA). Antibodies used for flow cytometry were all purchased from eBioscience (USA). Milli-Q Ultrapure water was used in all experiments.

*Preparation of PCL and PCL@MnO_x_ (PM) Scaffolds*: The PCL nanofibrous scaffold was fabricated by electrospinning.^[^[[1]](#endnote-1)^]^ First, PCL was dissolved in TFE at a concentration of 10 w/v% to prepare the electrospinning PCL solution. The prepared electrospinning solution was pumped out through a blunt needle at a flow rate of 1 mL/h, and then a high voltage of 17 kV was applied between the needle and a piece of aluminum foil coated on a rotating mandrel collector (500 rpm). After electrospinning, the obtained fiber scaffolds were dried in the fume hood to completely evaporate the residual solvent.

The PCL scaffold was then immersed in a DA solution (1 mg/mL in 10 mM ethanol-Tris buffer (v/v = 1:1), pH = 8.5) at 37 ^o^C for 6 h. Afterward, the scaffold was washed with deionized water for three times and dried in a stream of nitrogen gas. Then, the scaffold was added into 2 mL of 600 μg/mL KMnO_4_ solution at 37 ^o^C for 8 h. The PM scaffold was finally taken out and washed with deionized water for 5 times and dried for future use. For the preparation of PM scaffolds with different amounts of MnO_x_, the preparation processes are similar except that the concentration of KMnO_4_ was adjusted to 200 and 1000 μg/mL, respectively.

*Characterization:* The morphology of the scaffolds was observed by scanning electron microscopy (SEM, Zeiss, SUPRA 55) and transmission electron microscopy (TEM, FEI Tecnai G2 F30). A contact angle analyzer (OCA25, Data Physics) was applied to measure the static water contact angles (WCA) of the fibrous scaffolds. X-ray photoelectron spectroscopy (XPS, Kratos, AXIS-Hsi) was used to determine the chemical compositions of the fibrous scaffolds surface.

*Degradation Behavior of the MnO_x_ Nanozymes within the PM Scaffold*: The PM scaffold (20 × 20 mm^2^) was weighed and immersed in 10 mL of PBS with or without 1 mM GSH. All the samples were placed in a constant temperature shaker (37 °C) at a speed of 100 r/min. At predetermined timepoints of 0, 3, 5, 7, 10, and 14 days, the scaffolds were washed with deionized water and dried for 24 h and weighed. The degradation behavior was estimated from the weight loss of scaffolds.

*ROS Detection*: Different scaffolds (20 × 20 mm^2^) were immersed into 25.0 mM NaHCO_3_/5% CO_2_ buffer solution with or without 2 mM GSH for 24 h. Subsequently, the scaffold was removed, and 10 μg/mL MB and 10 mM H_2_O_2_ were added and incubated at 37 ^o^C for 15 min. The absorption spectra of MB were recorded by UV−vis spectrophotometer (Shimadzu, UV-2600, Japan). The generation of ·OH was also measured on an ELEXSYS-II E500 electron spin resonance (ESR) spectrometer (Bruker, Germany) using DMPO as the spin trapping agent.

*Detection of O_2_ Generation*: Different scaffolds (20 × 20 mm^2^) were immersed into H_2_O_2_ solution (10 mL, 0.5 mM). The concentration of dissolved O_2_ in the solution produced by scaffolds was recorded by a portable dissolved oxygen meter (INESA, China, JPSJ-605F).

*Cell Culture*: L929, RAW264.7, DC2.4, and 4T1 cell lines were provided by American Type Culture Collection. Luciferase-labeled 4T1 cells (4T1-Luc) were a gift from Professor X. J. Liang at National Center for Nanoscience and Technology. L929 and DC2.4 cells were cultured in Roswell Park Memorial Institute (RPMI) 1640 medium, containing 10% fetal bovine serum (FBS) and 1% penicillin/streptomycin. RAW264.7, 4T1, and 4T1-Luc cells were cultured in Dulbecco’s modified eagle medium (DMEM) containing 10% FBS and 1% penicillin/streptomycin. All cells were cultured at 37 °C with 5% CO_2_.

*Cytotoxicity Assay*: To evaluate the cytotoxicity of various scaffolds in L929, RAW264.7, DC2.4 and 4T1 cells, the cells were seeded in 24-well plates (5×10^4^ cells/well), followed by additional 24 h of incubation with different scaffolds (10 × 10 mm^2^). The cell viability was determined by MTT assay.

*In Vitro Assessments of Macrophage Polarization*: RAW264.7 cells were seeded in 6-well plates (5×10^5^ cells/well) and treated with IL-4 (20 ng/mL) for 24 h to induce M2 polarization. Subsequently, M2 macrophages were incubated with different scaffolds (20 × 20 mm^2^) for another 24 h. Afterwards, the cells were harvested and stained with anti-CD86-APC and anti-CD206-PE antibodies, followed by flow cytometry analysis (BD Accuri C6 plus, USA).

*In Vitro BMDC Stimulation*: BMDCs were extracted according to previous methods.^[^[[2]](#endnote-2)^]^ The obtained BMDCs were seeded in 6-well plates (1×10^6^ cells/well) and incubated with different scaffolds (20 × 20 mm^2^) for 24 h. To detect the DC stimulation, BMDCs were stained with anti-CD11c-FITC, anti-CD80-PE and anti-CD86-APC antibodies and were analyzed by flow cytometry.

*Intracellular ROS Detection*: 4T1 cells were seeded in confocal dishes at a density of 1 × 10^5^ cells per well overnight. Then the cells were treated with different scaffolds (10 × 10 mm^2^) for 24 h. After that, the cells were stained with 1 mL of DCFH-DA solution (10 μM in serum-free medium) in the dark and incubated for 30 min. Finally, the cells were washed and imaged using confocal laser scanning microscope (CLSM, Leica SP8).

*Characterization of Immunogenic Cell Death* *In Vitro*: For immunofluorescence analysis of CRT expression, 4T1 cells were seeded in confocal dishes at a density of 1 × 10^5^ cells per well overnight. Then the cells were treated with different scaffolds (10 × 10 mm^2^) for 24 h. After incubation, cells were washed and fixed with 4% paraformaldehyde for 10 min at room temperature and further incubated with anti-calreticulin antibody (Beyotime) for 2 h. Then, 4T1 cells were further incubated with Cy3-conjugated secondary antibody (Beyotime) for 1 h. Finally, the cells were stained with DAPI for 5 min and observed by CLSM.

The release of high mobility group box 1 protein (HMGB1) and adenosine triphosphate (ATP) was examined by the HMGB1 enzyme-linked immunosorbent assays (ELISA) and Enhanced ATP assay kit, respectively. Briefly, 4T1 cells in 24-well plates were treated with different scaffolds (10 × 10 mm^2^) for 24 h. Then, the supernatants were collected and centrifuged at 12000 g for 5 min at 4 ^o^C. The release of HMGB1 and ATP in the cell supernatant was detected by the HMGB1 ELISA Kit (Elabscience, China) and Enhanced ATP assay kit (Beyotime, China) according to the manufacturer’s protocols.

*Intracellular Hypoxia Detection*: 4T1 cells were seeded in confocal dishes at a density of 1 × 10^5^ cells per well overnight. Then the cells were treated with different scaffolds (10 × 10 mm^2^) for 24 h. After incubation, cells were washed and fixed with 4% paraformaldehyde for 10 min at room temperature and further incubated with anti-HIF-1*α* antibody for 2 h. Then, 4T1 cells were further incubated with Alexa Fluor 488-conjugated secondary antibody (Beyotime) for 1 h. Finally, the cells were stained with DAPI for 5 min and observed by CLSM.

*Postsurgical Tumor Models and Anti-recurrence Efficacy*: Female Balb/c mice (6 weeks) were obtained from Vital River Laboratory Animal Technology Co., Ltd. (Beijing, China). Animal experiments were approved by the Animal Ethics Committee of China-Japan Friendship Hospital (Beijing, China) and performed under legal protocols. To study the anti-recurrence efficacy of scaffolds in vivo, 1 × 10^6^ of 4T1 or 4T1-Luc cells were inoculated subcutaneously in the right flank of female Balb/c mice on day 0. On day 12, when the tumor volume reached around 300 mm^3^, mice were randomly divided into four groups for different treatments: (1) Control (only surgery); (2) PCL (implantation of PCL scaffolds); (3) PP (implantation of PP scaffolds); (4) PM (implantation of PM scaffolds). Approximately 90% of the total tumors was surgically removed. Subsequently, various scaffolds (30 × 30 mm^2^) were subsequently implanted into the resection cavity. The wound was sutured by the Autoclip wound clip system with 7-mm nails. The tumor volume and body weight of all mice were recorded every other day. The tumor volume was measured with a vernier caliper and calculated using the following formula: tumor volume (mm^3^) = length × (width)^2^ × 0.5. In vivo bioluminescence imaging was used to track tumor burden in real time. D-Luciferin (15 mg/mL, 100 μL per mouse) was intraperitoneally injected into each mouse eight minutes before being anesthetized with isoflurane. The mice were defined as dead when the tumor volume reached 1500 mm^3^. Survival rates from the day of tumor inoculation (day 0) to day 60 were assessed.

*Flow Cytometry Analysis*: To investigate the immune responses in vivo, the other two batches of mice were treated aforementioned and sacrificed on day 19 and day 26. Scaffolds, tumors, inguinal lymph nodes, and peripheral blood were collected for the analysis of immune cells by flow cytometry. Additionally, the proinflammatory cytokines TNF-*α* and IFN-*γ* in serum were detected using the corresponding ELISA Kits (Dakewe Biotech, China).

For DC maturation analysis, the inguinal lymph nodes of mice were gathered and filtered to gain single-cell suspension. Then, the cell suspensions were blocked with 2% FBS and stained with anti-CD11c-FITC, anti-CD80-PE, and anti-CD86-APC antibodies for flow cytometry assay.

Tumors obtained from mice were digested by collagenase IV, hyaluronidase, and DNase I (Solarbio, China) to obtain the single-cell suspension and filtered through 70 μm filters. Thereafter, the cell suspensions of tumors were collected and blocked with 2% FBS and diluted to 1×10^7^ cells/mL. For T cell activation analysis, the cell suspensions were stained with anti-CD3-FITC, anti-CD4-PE, and anti-CD8a-APC antibodies. For TAMs analysis, the cells were stained with anti-CD11b-FITC, anti-F4/80-PerCP-Cy5.5, anti-CD86-APC, and anti-CD206-PE antibodies. For MDSCs analysis, the cells were stained with anti-CD45-PE-Cy7, anti-CD11b-FITC, and anti-Gr-1-PE antibodies. For Tregs analysis, the cells were first stained with anti-CD3-FITC, anti-CD4-PE, and anti-CD25-APC antibodies and then permeabilized with 100 μL of Fixation/permeabilization buffer (Invitrogen, USA) and stained with anti-Foxp3-PE-Cy5.5 antibody. Finally, the cells were collected and analyzed by flow cytometry. At the same time, the population of T cells in spleens were also examined by flow cytometry.


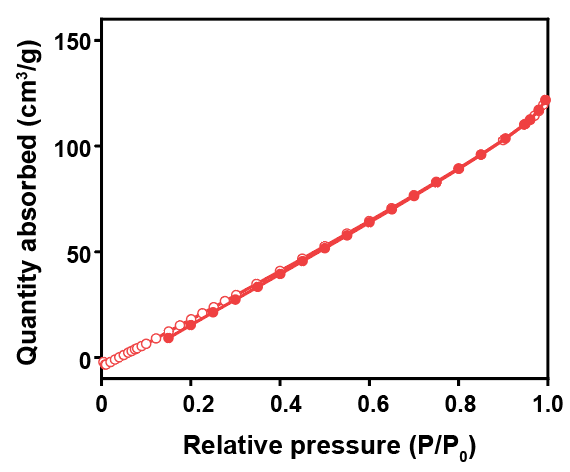


Figure S1. Nitrogen adsorption-desorption isotherms of the PM scaffold.


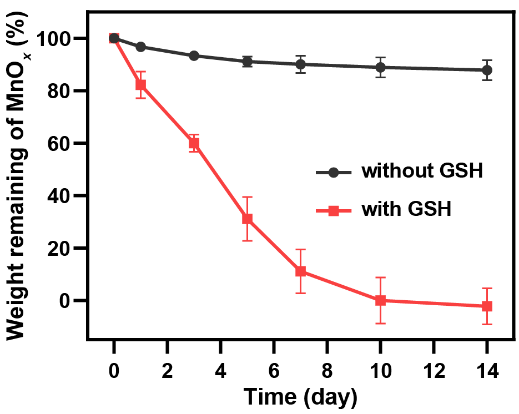


Figure S2. Biodegradation of the MnO*_x_* nanozymes in the PM scaffold at 37 °C with or without 1 mM GSH in PBS solution. (mean ± SD, n = 3)


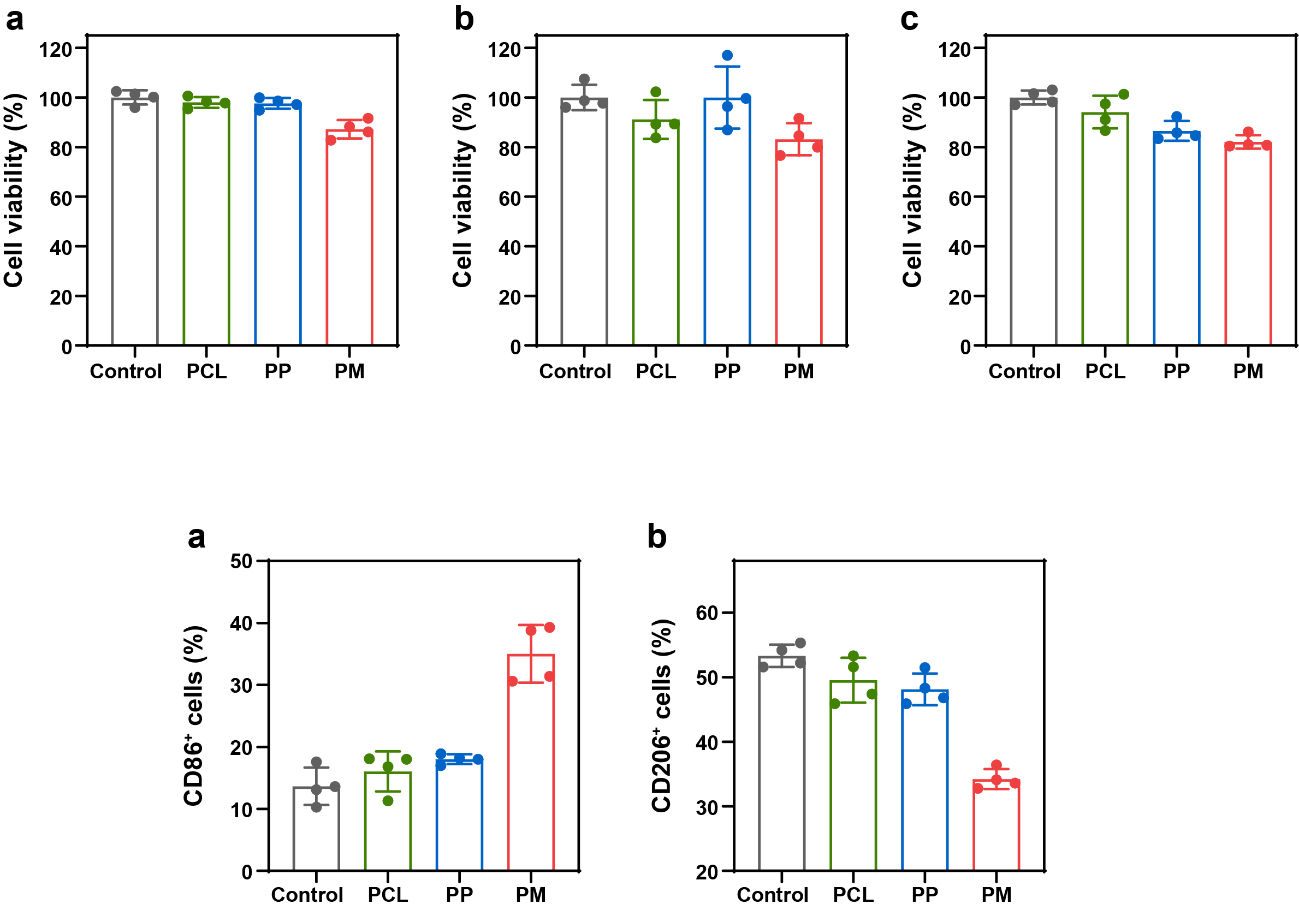


**Figure S3.** Viability of (a) L929, (b) RAW264.7, and (c) DC2.4 cells after different treatments (mean ± SD, *n* = 4).


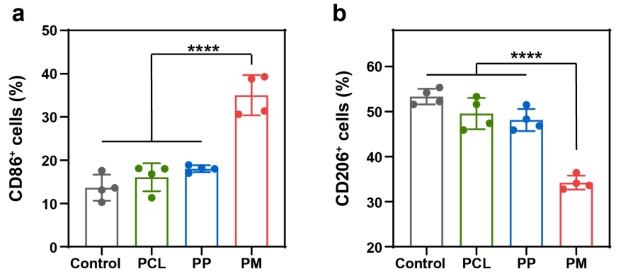


**Figure S4.** Quantitative analysis of (a) CD86 (M1 macrophage marker) and (b) CD206 (M2 macrophage marker) on RAW264.7 cells after incubation with various scaffolds by flow cytometry (mean ± SD, *n* = 4). One-way ANOVA followed by Tukey’s test. ^****^*p* < 0.0001


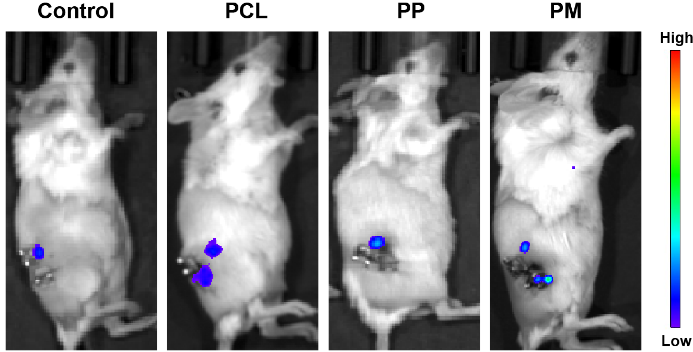


**Figure S5.** In vivo bioluminescence imaging of 4T1 tumor-bearing mice 1 d after surgery.


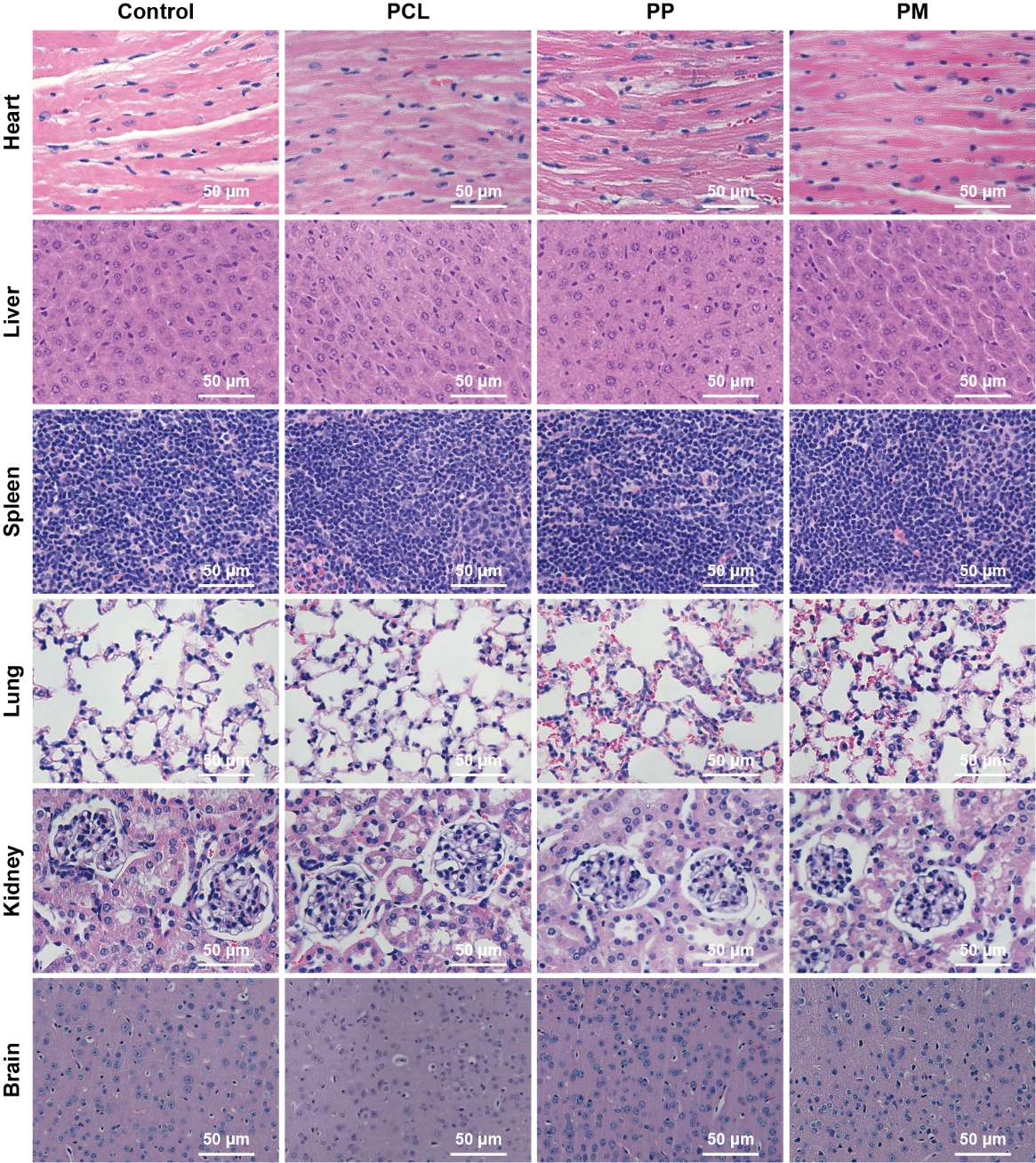


**Figure S6.** H&E staining of main organs (heart, liver, spleen, lung, kidney, and brain) in mice after different treatments.


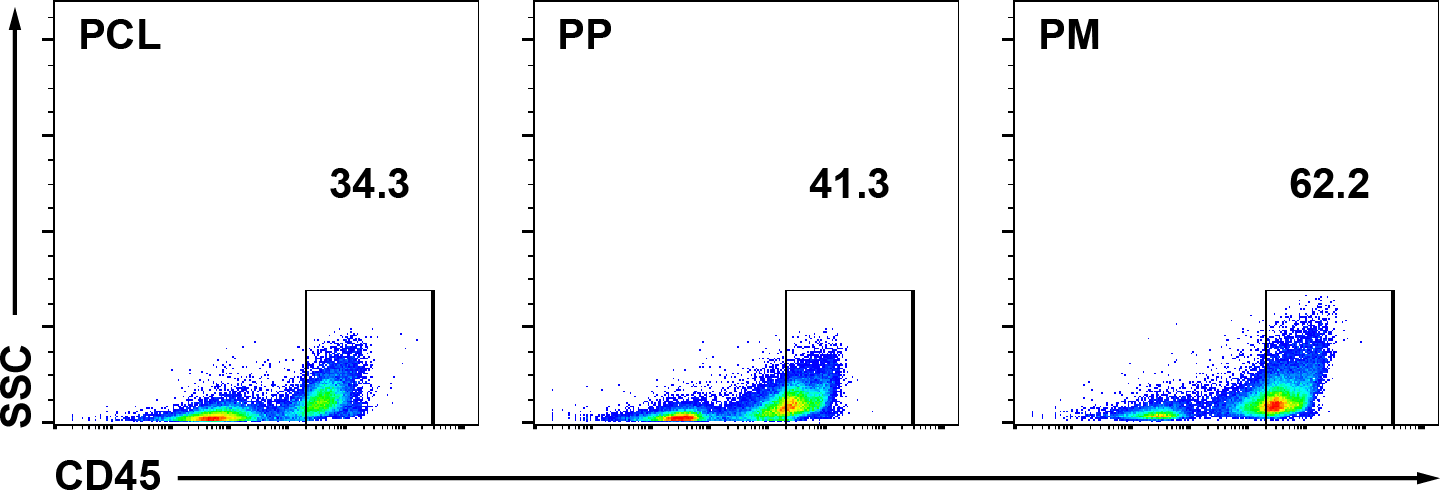


**Figure S7.** Representative flow cytometric analysis of CD45^+^ cells in infiltrated cells in different scaffolds 7 days after implantation.


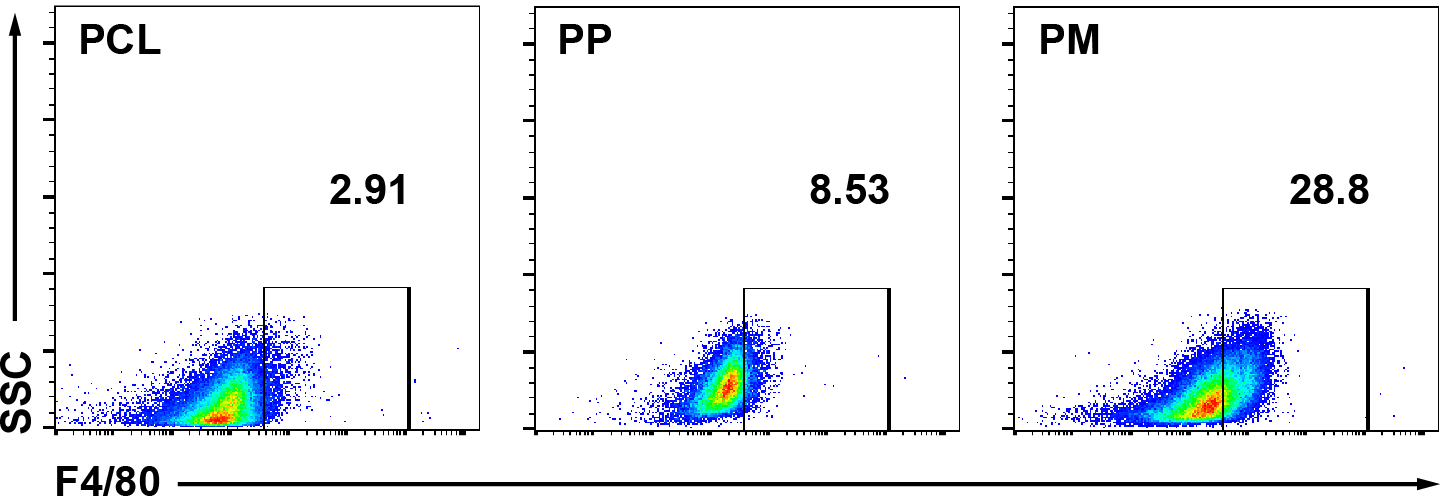


**Figure S8.** Representative flow cytometric analysis of F4/80^+^ cells in infiltrated cells in different scaffolds 7 days after implantation.


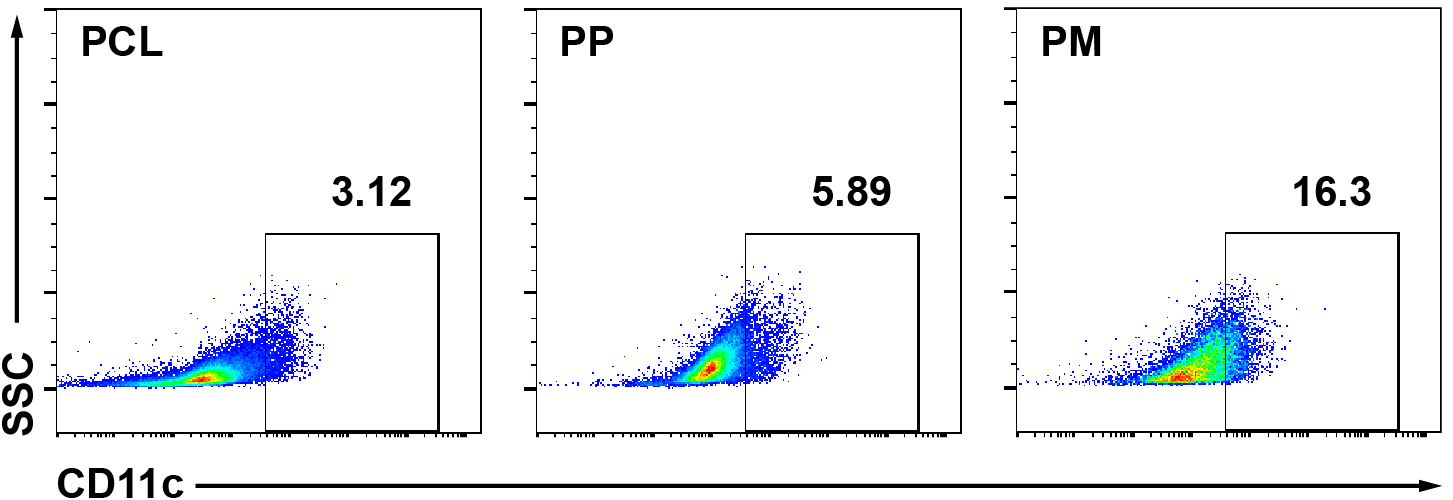


**Figure S9.** Representative flow cytometric analysis of CD11c^+^ cells in infiltrated cells in different scaffolds 7 days after implantation.


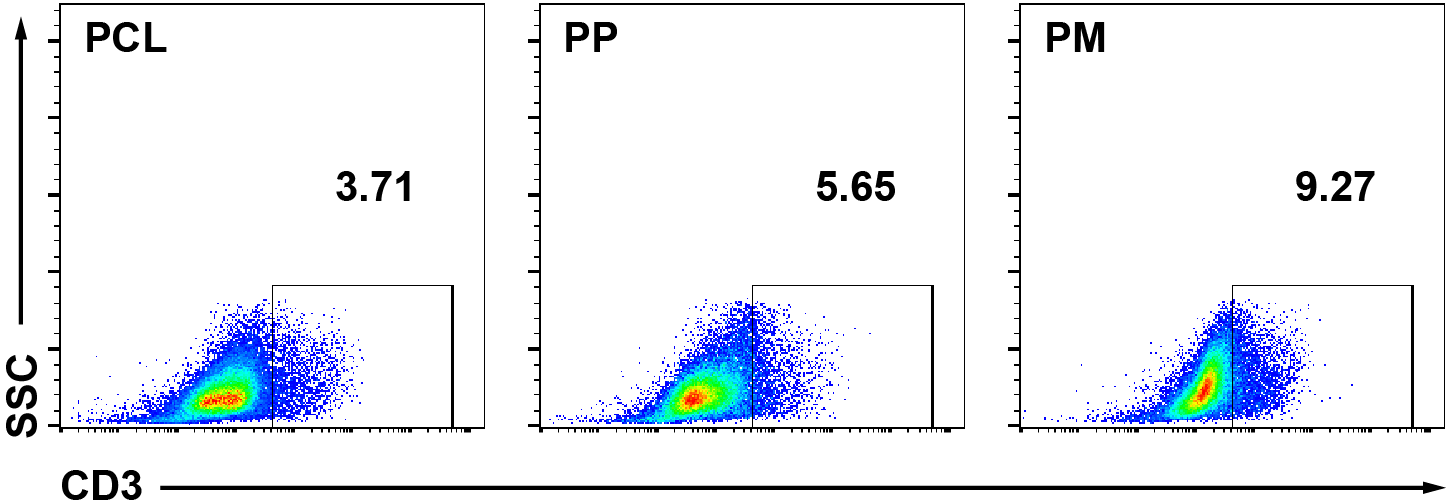


**Figure S10.** Representative flow cytometric analysis of CD3^+^ cells in infiltrated cells in different scaffolds 7 days after implantation.


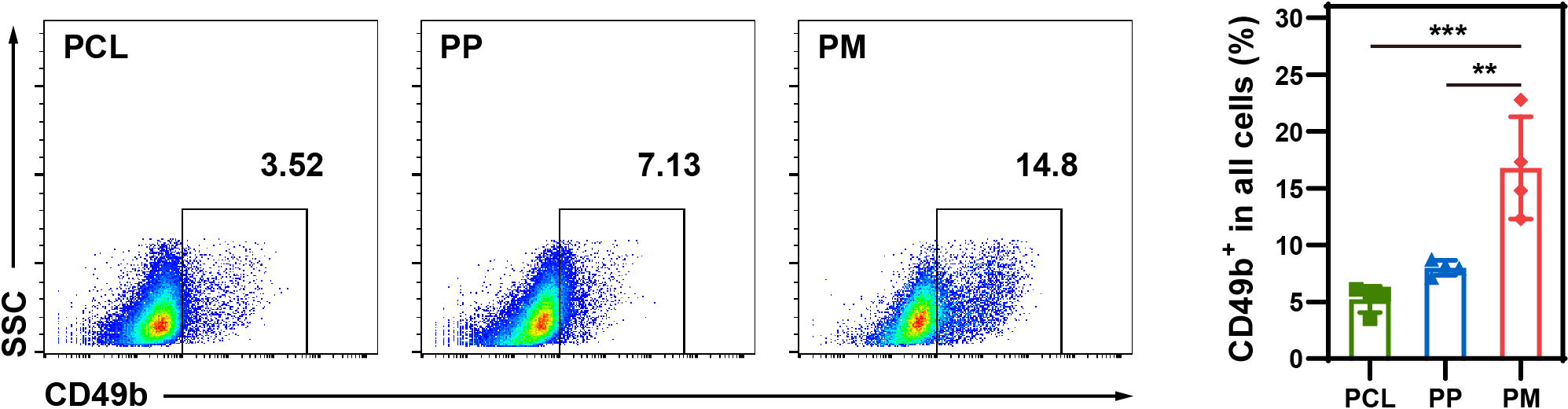


**Figure S11.** Flow cytometric analysis of CD49b^+^ cells in infiltrated cells in different scaffolds 7 days after implantation (mean ± SD, *n* = 4). One-way ANOVA followed by Tukey’s test. ^**^*p* < 0.01, ^***^*p* < 0.001.

**References**

1. [] X. Zhang, Q. Li, L. Li, et al., “Bioinspired Mild Photothermal Effect-Reinforced Multifunctional Fiber Scaffolds Promote Bone Regeneration,” *ACS Nano* 17 (2023): 6466–6479, https://doi.org/10.1021/acsnano.2c11486. [↑](#endnote-ref-1)
2. [] K. Inaba, M. Inaba, N. Romani, et al., “Generation of Large Numbers of Dendritic Cells from Mouse Bone Marrow Cultures Supplemented with Granulocyte/Macrophage Colony-Stimulating Factor,” *Journal of Experimental Medicine* 176 (1992): 1693–1702, https://doi.org/10.1084/jem.176.6.1693. [↑](#endnote-ref-2)
